# Complication experience during pregnancy and place of delivery among pregnant women: a cross-sectional study

**DOI:** 10.1186/s12884-023-05390-2

**Published:** 2023-03-11

**Authors:** Bekelu Teka Worku, Misra Abdulahi, Merertu Tsega, Birtukan Edilu, Rahma Ali, Mahilet Berhanu Habte, Samira Awel, Masrie Getnet, Yabsira Melaku, Radiet Kidane, Abonesh Taye, Meseret Tamirat

**Affiliations:** 1 Department of Population and Family Health, Jimma University, Jimma, Ethiopia; 2 Department of Nursing, Jimma University, Jimma, Ethiopia; 3 Department of Biostatistics, Jimma University, Jimma, Ethiopia; 4 Department of Nutrition and Dietetics, Jimma University, Jimma, Ethiopia

**Keywords:** Pregnancy complication, Place of delivery, Pregnant women, Cross-sectional study

## Abstract

**Background:**

Unlike other causes such as abortion, obstetric complications like hemorrhage, and hypertensive disorders of pregnancy, which are difficult to resolve for women who give birth out of health facilities are persisted or increased to be the cause of maternal mortality in Ethiopia. Direct obstetric complications resulted in the crude direct obstetric case fatality rate in this country. This study aimed to assess the relationship between Complication Experience during Pregnancy and Place of Delivery among Pregnant Women.

**Method:**

A community-based cross-sectional study was conducted to assess the baseline information as a part of a randomized control trial study. The sample size that was calculated for the cohort study with the assumptions to detect an increase in a minimum acceptable diet from 11 to 31%, with 95% CIs and 80% power, an intra-cluster correlation coefficient of 0·2 for a cluster size of 10 was used for this study. Statistical analysis was done using SPSS version 22.

**Result:**

The prevalence of self-reported pregnancy-related complications and home delivery were 79(15.9%, CI; 12.7–19.1) and 46.90% (95%CI; 42.5–51.1) respectively. Women who did not face vaginal bleeding were five times AOR 5.28(95% CI: 1.79–15.56) more like to give birth at home than those who faced this problem. Women who did not face severe headache were nearly three AOR 2.45(95%CI:1.01–5.97) times more like to give birth at home.

**Conclusion:**

This study concluded that home delivery was high among the study participants whereas pregnancy-related complications such as vaginal bleeding and severe headache were identified as protective factors for facility delivery. Hence, the researchers recommended the incorporation of “storytelling” into the existing health extension program packages to improve facility delivery which shall be applied after the approval of its effectiveness by further research.

## Background

Relating to maternal health, the recent focus of the global health agenda has expanded beyond the survival of women and their babies to ensuring they thrive and achieve their full potential for health and well-being [1]. In line with this, the time of child delivery is critical for both women and babies, as the risk of morbidity and mortality could increase considerably if complications happen [1, 2].

In less developed countries, maternal health intervention targets mostly women who can have complications during pregnancy or have other concerning issues such as distance from health facilities [3, 4]. However, evidence shows that women without complications are also experiencing maternal death [1]. For this matter, World Health Organization (WHO) is recommending good-quality and evidence-based intrapartum care for all women irrespective of the setting or level of health care with the importance of the development of relevant national and local level health policies and clinical protocols [1].

Unlike other causes such as abortion, obstetric complications like obstructed labor/uterine rupture (36%), hemorrhage (22%), and hypertensive disorders of pregnancy (19%) that are difficult to resolve for women who give birth out of health facilities are persisted or increased to be the cause of maternal mortality in Ethiopia [3, 5]. Direct obstetric complications resulted in the crude direct obstetric case fatality rate of 0.64% in Ethiopia where hypertensive disorders (27.8%) and maternal haemorrhage (23.9%) are the two leading causes [6].

Despite the high prevalence of direct obstetric complications with high maternal death, and great government effort to increase skilled obstetric care [4, 7], low institutional delivery is persisted and has shown a decreasing trend over time in some places in Ethiopia [8, 9]. According to the data from the 2019 Ethiopian Mini Demographic and Health Survey analysis, the prevalence of institution/facility delivery was 48.58% [8]. A high fever during pregnancy (14%), severe headache (57.5%), vaginal bleeding (8.6%), blurred vision (24.2%), convulsion (10.8%), swollen hand/face (16.9%), unconsciousness (12.1%) and water breakage (7.3%) are common complications that Ethiopian women reported during pregnancy [10, 11].

Different factors affect institutional delivery in Ethiopia. Women with a higher educational level, having antenatal care follow-up, being urban residents, community media exposure, community antenatal care coverage, lower parity, and better educational status of the husband had higher odds of giving birth at a health facility [7–9, 12]. Based on these and related evidence, recommendations were being forwarded to increase institutional delivery and to contribute to saving the lives of women and newborns. However, no study had assessed the association between complications experienced during third-trimester pregnancy and place of delivery in Ethiopia.

In some settings, particularly in less developed areas too few interventions or too many interventions that the women do not need and adhere to are being provided for the purpose of improving facility delivery service quality and its utilization [1]. So, this study helps to forward specific and relevant interventions to increase facility delivery. Thus, it aims to determine the relationship between complications experienced during third-trimester pregnancy and place of delivery among pregnant women.

## Method

### Study setting and period

The study was conducted in two districts of Jimma Zone named, Dedo districts and Seka Chekorsa districts in Oromia region, Southwest Ethiopia. The districts have 36 Kebeles each with a total population of 237,844 in Dedo district and 296,440 in Seka Chekorsa district in 2013 E.C (Ethiopian calendar). Based on the 2013 E.C. report, there were 8486 and 9436 pregnant women in Dedo and Seka Chekorsa districts respectively. Dedo district has One hospital, eight health centers, and nine private clinics whereas Seka Chekorsa district has one public hospital, nine public health centers, and 16 private health facilities. All information was obtained from the zonal health bureau. The study was conducted from August 20 to 24/2022GC.

### Study design

A community-based cross-sectional study design was employed to collect the data.

### Study population

All pregnant women who were selected from the selected kebeles in the Dedo district and Seka Chokorsa district kebeles and who fulfil the inclusion criteria were the study population.

### Inclusion and exclusion criteria

The women were included based on the inclusion criteria set for the cohort study which includes pregnant women in the third trimester who have lived in the selected kebeles for not less than six months, and were willing to be visited by data collectors and supervisors after the child delivery. Similarly, exclusion criteria were serious illness (women diagnosed with hyperemesis) or clinical complications requiring hospitalization, twin pregnancy, or any child congenital abnormality identified by experts.

### Sample size determination and sampling procedure

Sample size that was calculated for the cohort study was used for this study. It was determined with the assumptions to detect an increase in a minimum acceptable diet from 11 to 31% [13], with 95% CIs and 80% power, an intra-cluster correlation coefficient of 0·2 for a cluster size of 10. It was calculated that 52 clusters were needed. Adding 20% of the sample size for loss to follow-up, the final sample size was 624 pregnant women (312 in intervention, and 312 in control groups). Being a part of baseline data, this study used the sample size calculated for the two groups as it is.

The cohort study was proposed on any pregnant women residing in the selected kebeles who fulfill the inclusion criteria. During the analysis of this baseline data, 98 women who were primigravida were excluded from the analysis since the objective of this study was on last delivery. To ensure the adequacy of the remaining number for sample size, the sample was calculated using single population proportion formula for the prevalence (15.9%) of pregnancy-related complications in Ethiopia [10] with the assumptions of a 95% confidence level, 5% degree of precision, and 10% non-response rate. Thus, n = [Zα/_2_]^2^*p*(1 − p)/d^2^= (1.96)^2^*0.159(1 − 0.159) / (0.05)^2^= 227. We used 497 sample which is greater than the minimum required sample.

### Participant Recruitment

The study areas were clustered by kebele. All third-trimester pregnant women residing in the selected kebeles were identified and enrolled in the study using the updated Health Extension Workers’ antenatal care logbook. Pregnant women were also identified through the one-to-five network to reduce the possibility of missing them. Then, all the identified pregnant women in their third trimester were enrolled in the study.

### Data collection tool, method, and personnel

The data collection tool was prepared from relevant literature [14–18] originally in English, translated into the local language Afan Oromo and back-translated by other language experts. The tool was tested on 5% of the sample calculated for cohort study and necessary measurement was taken before the actual data collection. Ten females, who had completed at least 10th grade were recruited, trained, and worked on data collection. The data were collected by these interviewers through a home-to-home face-to-face interview. The data collection process was strictly supervised by the research team and trained supervisors. Data collection was done using the KoboCollect mobile application.

### Data analysis

Statistical analysis was done using SPSS version 22. Descriptive statistics were used to summarize the characteristics of the participants. Bivariate logistic regression analysis was done for each variable with the outcome variable to select candidate variables at *p*-value < 0.25. Then, multivariate logistic regression analysis was done to control for possible confounding variables and to determine the presence of a statically significant association between the predictors and the outcome variable at *p*-value < 0.05 and AOR with 95% CI. Multicollinearity and model fitness was checked and out-ruled.

## Result

### Socio-demographic characteristics of the participants

All study participants were married and living with their partners. The mean age of the respondents was 26.78 (SD ± 4.88) years (Table 1).

**Table 1:** Distribution of Socio-demographic characteristics of participants

Variables	Categories	Frequency (Percent)
Residence of the participants (district)	Dedo district	254(42.7)
	Seka Chokorsa district	243(40.8)
Age of participants in years	18–19	9(1.85)
	20–24	136(27.4)
	25–29	164(33.0)
	30–34	126(25.4)
	35–39	59(11.9)
	40–44	3(0.6)
Educational status of the participants	No formal education	238(47.9)
	Primary school (1–8 grades)	163(32.8)
	Secondary school (9–12 grades)	94(18.9)
	College and above	2(0.4)
Ethnicity of the participants	Oromo	480(96.)
	Amhara	8(1.6)
	Other	9(1.5)
Religion of the participants	Muslim	477(96.0)
	orthodox	13(2.6)
	protestant	7(1.4)

### Obstetric and maternal health service-related characteristics of the participants

The mean duration of the last child delivery for which the history was asked was 36.54(SD ± 13.44) months (Table 2).

**Table 2:** Distribution of obstetric history of women who participated in the study

Variables	Categories	Frequency(percent)
How many times have the women given birth	Once	105(21.1)
	2 to 5 times	330(66.4)
	More than five times	62(12.5)
Women who had bad obstetric history at least once	Abortion	41(8.2)
	Stillbirth	36(7.2)
	Child death	56(11.3)
Age of the most recent child	Less than 24 months	47(9.5)
	24 to 60 months	433(87.1)
	Greater than 60 months	17(3.4)
Women who had attended antenatal care service at least once	Yes	385(77.5)
	No	112(22.5)
Frequency of antenatal care services used	One visit	13(3.4)
	2 to 3 visits	246(64.7)
	Four and above four visits	121(31.8)
Place of antennal care service	Health post	157(40.8)
	Health center	158(41.0)
	Private clinic	10(2.6)
	Hospital	60(15.6)
Time of antennal care service initiation in gestational age in month	1 to 3 months	74(19.2)
	4 to 6 month	265(68.8)
	7 to 9 month	36(9.4)
	Don’t remember exactly	10(2.6)

**Fig. 1:**
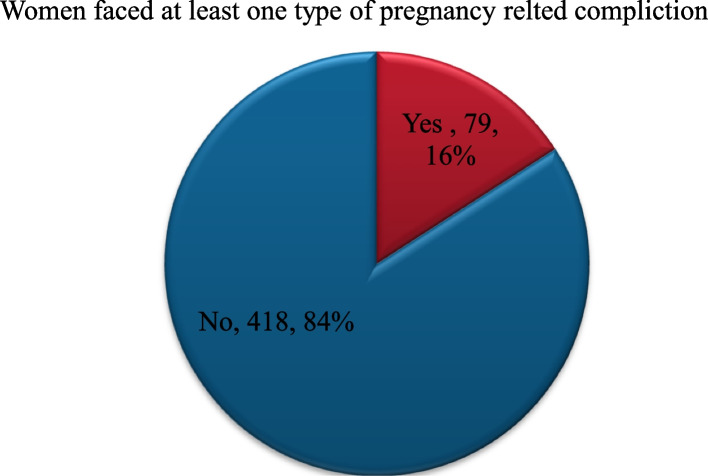
Prevalence of women who faced any type of self-reported pregnancy-related complication during pregnancy of their most recent child

About 15% 79(15.9%) of women had faced pregnancy-related complications during the pregnancy of their most recently delivered child (Fig. 1).

**Fig. 2:**
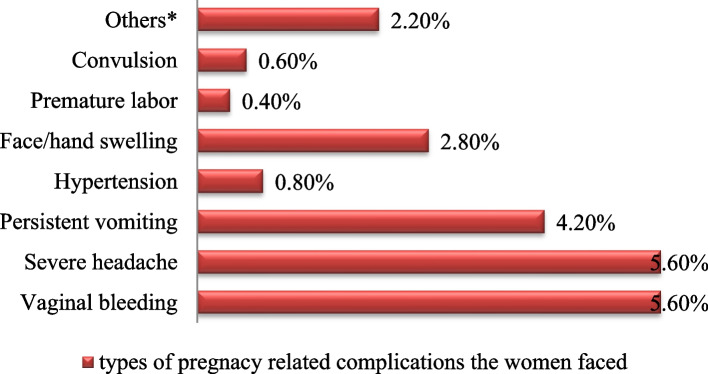
Distribution of types of self-reported pregnancy**-**related complications the women faced

Vaginal bleeding and severe headache were the most common pregnancy**-**related complications that the women who participated in the study reported facing the complications (Fig. 2).

### Place of delivery

**Fig. 3:**
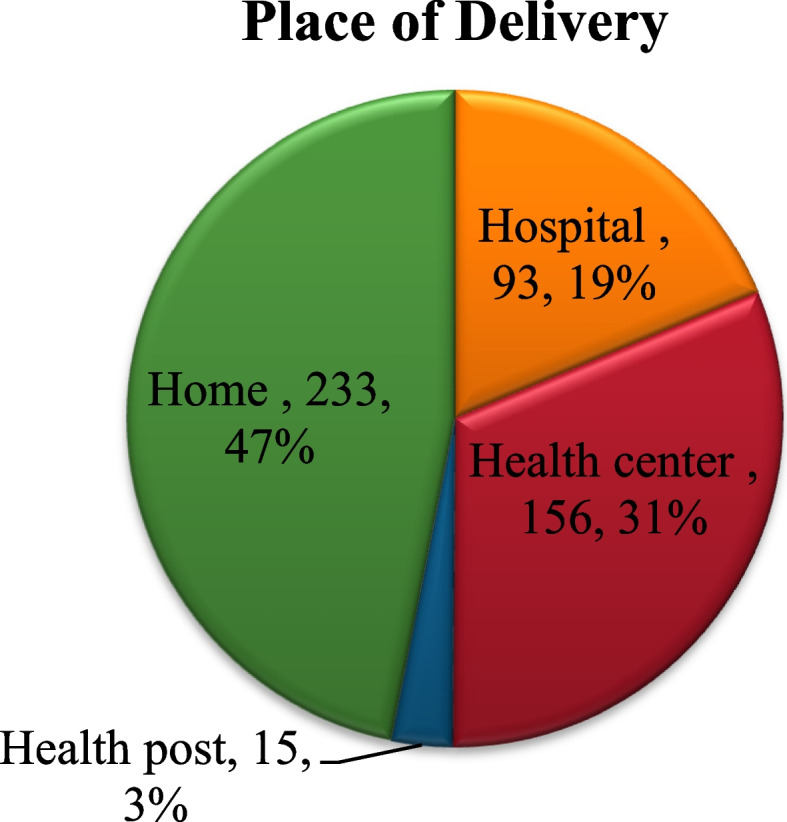
Distribution of place of child delivery among women who participated in the study

The majority of the women who participated in the study had given birth to their most recent delivery at home (Fig. 3).

### Relationship between complication experience during pregnancy and place of delivery

A large number of women who faced pregnancy-related complications during their most recent pregnancy had gave birth at home (Table 3).

**Table 3:** Self-reported pregnancy-related complications and place of delivery among women who participated in the study

	Options	Have you faced any pregnancy-related complications during your last pregnancy?		
		Yes	No	Total
Where did you give birth to your most recent child?	Hospital	21(26.6%)	72(17.2%)	93(18.7%)
	Health center	37(46.8%)	119(28.5%)	156(31.4%)
	Health post	3(3.8%)	12(2.9%)	15(3.0%)
	Home	18(22.8%)	215(51.4%)	233(46.9%)

### The logistic regression analysis result

In bivariate logistic regression, vaginal bleeding, severe headache, persistent vomiting, face/hand swelling, and others were identified as candidate variables for multivariate analysis from the seven pregnancy-related complications. Two pregnancy-related complications; vaginal bleeding with a *p*-value of 0.003 and severe headache with a *p*-value of 0.049 were significantly associated with the outcome variable in multivariate analysis. According to this result, women who did not face vaginal bleeding were five times AOR 5.28 (CI; 1.79–15.56) more like to give birth at home than those who faced this problem. Similarly, women who did not face severe headache were nearly three AOR 2.45 (CI; 1.01–5.97) times more like to give birth at home than those who faced the problem (Table 4).

**Table 4:** Logistic regression analysis output showing the association between pregnancy-related complications and place of delivery among the women who participated in the study

Variables	Categories	Where did you give birth to your last child		COR(95% CI)	AOR(95% CI)
		Health facility	Home		
Vaginal bleeding	Yes	24	4	1	1
	No	240	229	5.73(1.96, 16.76)	5.28(1.79, 15.56)*
Severe headache	Yes	21	7	1	1
	No	243	226	2.79(1.16, 6.69)	2.45(1.01, 5.97)*
Persistent Vomiting	Yes	15	6	1	1
	No	249	227	2.28(0.87, 5.97)	1.43(0.51, 4.04)
Face/hand swelling	Yes	4	0	1	1
	No	260	233	3.3(0.92, 12.10)	1.72(0.42, 6.10)
Other pregnancy complications	Yes	8	3	1	1
	No	256	230	2.40(0.63, 9.14)	2.37(0.61, 9.16)

*Shows the statistical significance of the association at *p*-value < 0.05

## Discussion

This study established an association between pregnancy-related complications and place of delivery among women who had at least one**-**time history of child delivery in two districts of Jimma Zone of Oromia in Ethiopia. A large number of respondents reported that they had faced pregnancy-related complications whereas; the most frequently reported complications were vaginal bleeding and severe headache. Home delivery was also high while women who did not face pregnancy**-**related complications were more likely to give birth at home.

The prevalence of self-reported pregnancy**-**related complications among the study participants was 79(15.9%, CI; 12.7, 19.1). This finding is consistent with the study finding 372 (15.9%) from Northwest Ethiopia [10]. In both studies, the finding is high. The high prevalence of pregnancy**-**related complications could be associated with the physical and social characteristics of the participants where young aged women were higher in this study and young age is a risk factor for pregnancy**-**related complications [19].

However, this self-reported pregnancy**-**related complications magnitude is lower than 27.4% (78/285) the study result from Uganda [20]. The discrepancy could be due to differences in the study setting, data collection time, and study method. Our study was a community-based study, using a self**-**reported approach among mothers who gave birth and that of Uganda is a facility**-**based study where they have a chance to review the records for missed information and this can reduce recall bias. Additionally, the time of data collection can also create a difference since our study is conducted a few years after delivery where there could be the effect of recall bias [21].

In our study, home delivery among the study participants was 46.90%( 95% CI; 42.5–51.1). This result is lower (88.3%) compared to the result of the study done in Northern Ethiopia [22] and the result (73.8%) of the study conducted in Ethiopia nationally [23]. This variation could be due to the difference in sample size where the study of Northern Ethiopia had a smaller sample size, and the national data is differently high. The disparity in study time might also matter as maternal health service utilization is increasing from time to time in Ethiopia [24].

On the other hand, the prevalence of home delivery in this study was higher than the finding of a pooled analysis of home delivery in East Africa 23.68% (95% CI:23.45–23.92) [25]. The high prevalence of home delivery in this study could be due to the effect of fear of COVID-19, and poor common client satisfaction in this country [26–28].

Facility delivery in our study was 53.1% (95% CI: 48.9–57.5). This outcome is higher compared to the result of a study conducted in the Afar region 35.2% (95% CI: 30.5–40.1) and Gurage zone of Southern Nations, Nationalities, and Peoples’ Region of Ethiopia (31%) [17, 18]. This difference could be due to the variation in the study setting, study period, and economical variation of the study participants. Accessibility to health institutions is more difficult in the two regions compared to our study setting [29].

However, the facility delivers in our case is lower than that of Waka town of South Nations and Nationalities of Ethiopia (89.4%), Woldia Town of Ethiopia (74.7%), and Boset Woreda, Central Ethiopia (60%) [14–16]. The lower magnitude of facility delivery in our study might be due to the nature of the study area where it was conducted majorly in the rural area and all maternal health service utilization in Ethiopia is lower in rural compared to urban [29].

### Association of pregnancy-related complications and place of child delivery

The logistic regression analysis result of this study identified that two of the self-reported pregnancy**-**related complications had a statistical association with facility delivery where the women who did not face vaginal bleeding and severe headache were much more likely to give birth at home compared to those who faced these complications.

The majority of the previously conducted study in Ethiopia regarding identifying factors associated with place of delivery focused on maternal**-**related factors such as socio-economic and socio-cultural factors, and facility-related factors like distance and quality of service. Also, several efforts have been made to resolve these factors by the Ethiopian government to increase maternal health services utilization; particularly to increase institutional delivery service utilization. Regardless of all these efforts, skilled delivery and facility delivery utilization remained very low in this country despite the persistently high maternal death. Our study focused on the association between pregnancy complications and place of delivery which can contribute to a new way of investigating the challenges for improvement of maternal health service utilization.

The result indicated that women who did not face vaginal bleeding were five times AOR 5.28 (95% CI: 1.79–15.56) more like to give birth at home than those who faced this problem. Likewise, women who did not face severe headache were nearly three AOR 2.45 (95% CI: 1.01–5.97) times more like to give birth at home than their counter groups. Similar to these results, different studies had identified that pregnancy-related complications have a significant and positive association with child delivery in health facilities.

Pregnancy complications recognized at antenatal care was identified (OR 2.4, 95% CI 1.3–4.6) as a factor associated with health facility delivery [30] in the Philippines. Moreover, a systematic review conducted on institutional delivery in Ethiopia revealed that women who encountered problems during pregnancy (OR = 2.83, CI = 4.54, 7.39) had a higher chance of giving birth in health facilities [31]. Further, a study in Southwest Ethiopia (AOR = 3.86, 95% CI: 2.67– 7.29) and Benishangul Gumuz, West Ethiopia (AOR = 1.95, 95% CI: 1.01, 4.23,) indicated the experience of pregnancy**-**related danger signs had a positive association with institutional delivery.

The possible explanation for the association might be that facing complications during pregnancy initiates the women for the utilization of different maternal health services such as prenatal which could help them with birth preparedness and complication readiness that in turn can increase institutional delivery [27, 32]. Women who had faced complications can have more practical experience in life**-**threatening conditions than those who did not which can motivate them to give birth under the supervision of professionals who can help them during an emergency in case it happens. In addition, experiencing complications can make women seek health care services during pregnancy and they can have a chance of being recommended facility delivery by health professionals.

## Conclusion and recommendation

This study concluded that home delivery is high among the study participants whereas the happening of pregnancy-related complications such as vaginal bleeding and severe headache were identified as protective factors for facility delivery. Hence, the researchers recommended the incorporation of “storytelling” in awareness creation from the experience of pregnant women who had experienced a complication during pregnancy into the existing health extension program packages in Ethiopia to improve facility use. However, it shall be applied after the approval of its effectiveness by further research.

### Limitations of the study

This study was conducted among women who gave birth in a previous period which extends to six years because we could not exclude mothers with a long delivery time as it is not ethical to exclude them from the interventions. This study also did not identify the reason for home delivery due to fear of recall bias.

## Data Availability

all data used to prepare this article is available within the article. If further is needed, the interested person can contact the corresponding author via bekelut23@gmail.com or bekelutybh21@gmail.com on a reasonable base.
